# Object detection algorithm for eggs of *Pomacea canaliculata* in a paddy field environment

**DOI:** 10.3389/fpls.2025.1683763

**Published:** 2025-11-13

**Authors:** Guang Qi Wang, Jing He, Rui Ning Hu, Dian Li, Gang Liu

**Affiliations:** 1 College of Geography and Planning, Chengdu University of Technology, Chengdu, China; 2 College of Earth and Planetary Sciences, Chengdu University of Technology, Chengdu, China

**Keywords:** eggs of *Pomacea canaliculata*, omni-dimensional dynamic convolution, slim-neck, receptive-field attention, YOLOv8

## Abstract

As an invasive species in China, *Pomacea canaliculata* severely impacts crop quality and yield, necessitating effective monitoring for food security. To address the challenges in detecting its eggs in paddy fields—including feature contamination, stem and leaf occlusion, and dense targets—we propose an enhanced YOLOv8n-based algorithm. The method introduces omni-dimensional dynamic convolution (ODConv) in the backbone network to improve target feature extraction, constructs a Slim-neck structure to optimize feature processing efficiency, and designs a receptive-field attention head (RFAHead) for detection refinement. Experimental results demonstrate that the improved model achieves 3.3% and 4.2% higher mAP@0.5 and mAP@0.5:0.95 than the original YOLOv8. It outperforms Faster R-CNN, YOLOv3-tiny, YOLOv5, YOLOv6, YOLOv7-tiny, YOLOv9-t, YOLOv10n, and YOLOv11n by 18.2%, 12.4%, 5.2%, 10.8%, 11.6% 5.0%, 3.8%, and 3.4% in mAP@0.5 and 20.6%, 17.5%, 8.1%, 15.6%, 16.1%, 7.0%, 7.7%, and 6.5% in mAP@0.5:0.95, respectively. Visual analysis confirms enhanced recognition of small and occluded targets through improved feature learning. This model enables accurate and rapid detection of *Pomacea* eggs in rice fields, offering technical support for invasive species control.

## 1 Introduction

*Pomacea canaliculata* has emerged as a highly invasive alien species in China, exhibiting remarkable adaptability and fecundity. This mollusk has established populations across 18 provincial-level administrative divisions, including municipalities and autonomous regions, from Sichuan to Fujian provinces. Its invasion poses significant threats to various sectors, including agriculture, forestry, animal husbandry, and aquaculture, while it concurrently jeopardizes ecological integrity and public health in the affected regions. The species’ high reproductive output directly impacts crop growth parameters, leading to substantial reductions in both yield quantity and quality (Zhang et al., 2017; Yin et al., 2022; Zhuo et al., 2022). Accurate and efficient detection of *P. canaliculata* and its egg masses is a fundamental prerequisite for investigating invasion mechanisms and dispersal dynamics. Such capabilities are critical for designing evidence-based prevention and control strategies (Jiang et al., 2024). Current diagnostic approaches for crop pests predominantly rely on manual identification or machine learning-based detection systems. While manual interpretation achieves high accuracy and machine learning enhances operational efficiency, these methods present notable limitations. Their implementation requires specialized expertise along with extensive training datasets; moreover, models often exhibit limited generalizability across heterogeneous field conditions (Gao et al., 2010).

The integration of artificial intelligence into agricultural diagnostics has accelerated markedly, with convolutional neural networks (CNNs) emerging as pivotal tools for rapid identification of crop diseases and pests. These deep learning architectures are driving paradigm shifts in precision agriculture through enhanced operational scalability and decision-making efficiency (Wei, 2017). Pioneering work by Zhang (2018) demonstrated the synergistic application of CNNs with Otsu threshold optimization, achieving 95% mean average precision (mAP) in classifying five prevalent potato pathogens—a critical advancement for field-deployable diagnostic systems. Subsequent innovations by Tetila et al. (2020) developed a robust detection architecture combining ResNeXt-50 with region-based fully convolutional network (R-FCN) feature extraction, enabling precise localization and classification of nine distinct tomato plant disorders with 85.98% mAP accuracy. In parallel, Singh et al. (2019) engineered a multi-column CNN through strategic modifications to AlexNet’s convolutional layers, attaining state-of-the-art performance (97.13% mAP) on anthracnose-infected mango datasets, thereby establishing a benchmark for tropical fruit disease diagnostics.

The YOLO algorithm series has revolutionized computational object detection through its computationally efficient architecture, achieving real-time processing speeds that are driving transformative applications in precision agriculture for crop disease and pest surveillance (Guo et al., 2022). In a seminal comparative study, Guo et al. (2021) systematically evaluated detection frameworks using convolutional feature extraction, benchmarking Faster R-CNN, SSD (single shot multibox detector), and YOLOv3 architectures across 2,500 annotated rice pathology images. Their analysis revealed YOLOv3’s superior performance, attaining 91.93% mAP in classifying five critical *Oryza sativa* pathogens—establishing new standards for field-ready diagnostic systems. Building on these advancements, Zhang et al. (2022) engineered an enhanced YOLOv5 variant through strategic integration of DenseNet connectivity patterns, attention-guided feature selection, and bidirectional feature pyramid networks (BiFPN). This hybrid architecture demonstrated exceptional precision in detecting pre-anthesis cotton bolls under complex field conditions, resolving long-standing challenges in phenological stage monitoring. Parallel developments by Liu et al. (2024) incorporated a Ghost module into YOLOv7’s backbone network, effectively eliminating feature redundancy while implementing a multi-scale fusion framework combining the convolutional block attention module (CBAM) and BiFPN. Their optimized system achieved 93.2% mAP in detecting six distinct *Prunus persica* pathogens, particularly enhancing recognition accuracy for submillimeter lesion patterns through spectral–spatial feature correlation. Huang et al. (2023) proposed a YOLO-EP model, which incorporated the Swin Transformer to achieve the interaction between local and global information and integrated the Efficient Channel Attention (ECA) mechanism into the network to prevent the loss of feature information within the network. For *P. canaliculata* eggs in paddy fields, the improved model achieved an mAP of 88.6%.

Owing to variations in crop growth environments and leaf density, automated pest and disease detection systems frequently encounter challenges including pronounced illumination disparities and mutual occlusion among plant organs. To mitigate these limitations, recent methodological innovations have employed generative adversarial networks (GANs) to reconstruct occluded target features through self-adversarial learning frameworks. An et al. (2021) developed generative adversarial networks for learning occluded features (GANLOF), a novel architecture that restores damaged feature representations in training samples, effectively addressing target occlusion issues. This approach demonstrated a 1.61% improvement in recognition accuracy for partially obscured targets compared to conventional methods. Building upon these advancements, Yan et al. (2022) integrated the convolutional block attention module (CBAM) with cross-scale feature fusion pyramid pooling into the YOLOv7 framework. Their proposed ACFP-YOLO algorithm significantly enhanced detection performance for occluded targets through optimized attention weighting and multi-scale feature aggregation. These methodological innovations have collectively advanced object detection capabilities in agricultural pest monitoring, particularly demonstrating applicability for *P. canaliculata* egg detection in complex paddy field ecosystems. The synergistic combination of feature reconstruction and attention-driven detection frameworks lays a foundation for robust agricultural surveillance systems operating under real-world field conditions.

The accurate detection of *P. canaliculata* eggs in submerged paddy field environments faces three critical challenges: illumination-induced luminance variations that degrade image clarity through dynamic water-surface reflections, frequent occlusions caused by submerged rice stalks and floating foliage, and diminished foreground–background contrast due to the eggs’ small size and visual similarity to organic debris. These factors collectively lead to feature contamination, where morphological signatures of egg clusters become indistinguishable from environmental noise. Existing object detection algorithms exhibit significant limitations in this context, including excessive model complexity, unresolved speed–accuracy trade-offs, inadequate resolution for small-size object discrimination, and degraded performance under partial-to-complete occlusion (Xu et al., 2023; Zhang et al., 2024).

To address the abovementioned problems, this paper makes improvements based on YOLOv8n and proposes an occlusion-resistant small target detection algorithm for *P. canaliculata* eggs in a paddy field environment. The main work of this algorithm is as follows:

1. Construct a dataset of *P. canaliculata* eggs in paddy fields under occlusion conditions and propose a target detection algorithm based on the YOLOv8n model to solve the problems of missed and false detections of *P. canaliculata* eggs in the paddy field environment.
2. Introduce ODConv into the backbone of the model. Employ a dynamic multi-dimensional attention mechanism to learn the complementary attention of the convolution kernel in all four dimensions of the kernel space, thereby improving the ability to extract features from egg images.
3. Incorporate the Slim-neck architecture into the neck of the model to build an efficient four-”neck” neural network, enabling the model to maintain high accuracy while reducing computational complexity and inference time.
4. Introduce the RFAHead at the detection head. By combining the processing of spatial attention and receptive-field features, it provides a new and more efficient way for the convolutional neural network to extract and process image features.

## 2 Materials and methods

### 2.1 Dataset preparation

The generalization and robustness of models are often influenced by the quality of the dataset. Therefore, in this study, a high-quality dataset of *P. canaliculata* egg images and samples in paddy fields was constructed. The study area is located in Yaodu Town, Qingbaijiang District, Chengdu City, Sichuan Province. The dataset was collected on-site and manually processed. Field data were captured using a DJI Phantom 4 Pro and handheld devices; the image resolution of DJI-acquired data is 5,742 × 3,648, while that of handheld device-acquired data is 3,648 × 2,736. The total number of collected original images was 969. To meet the diversity of the dataset and simulate the conditions of *P. canaliculata* eggs in different environments, data on *P. canaliculata* eggs were collected from different shooting angles in this study. The constructed dataset includes images of non-occluded eggs and occluded eggs.

**Figure 1:**
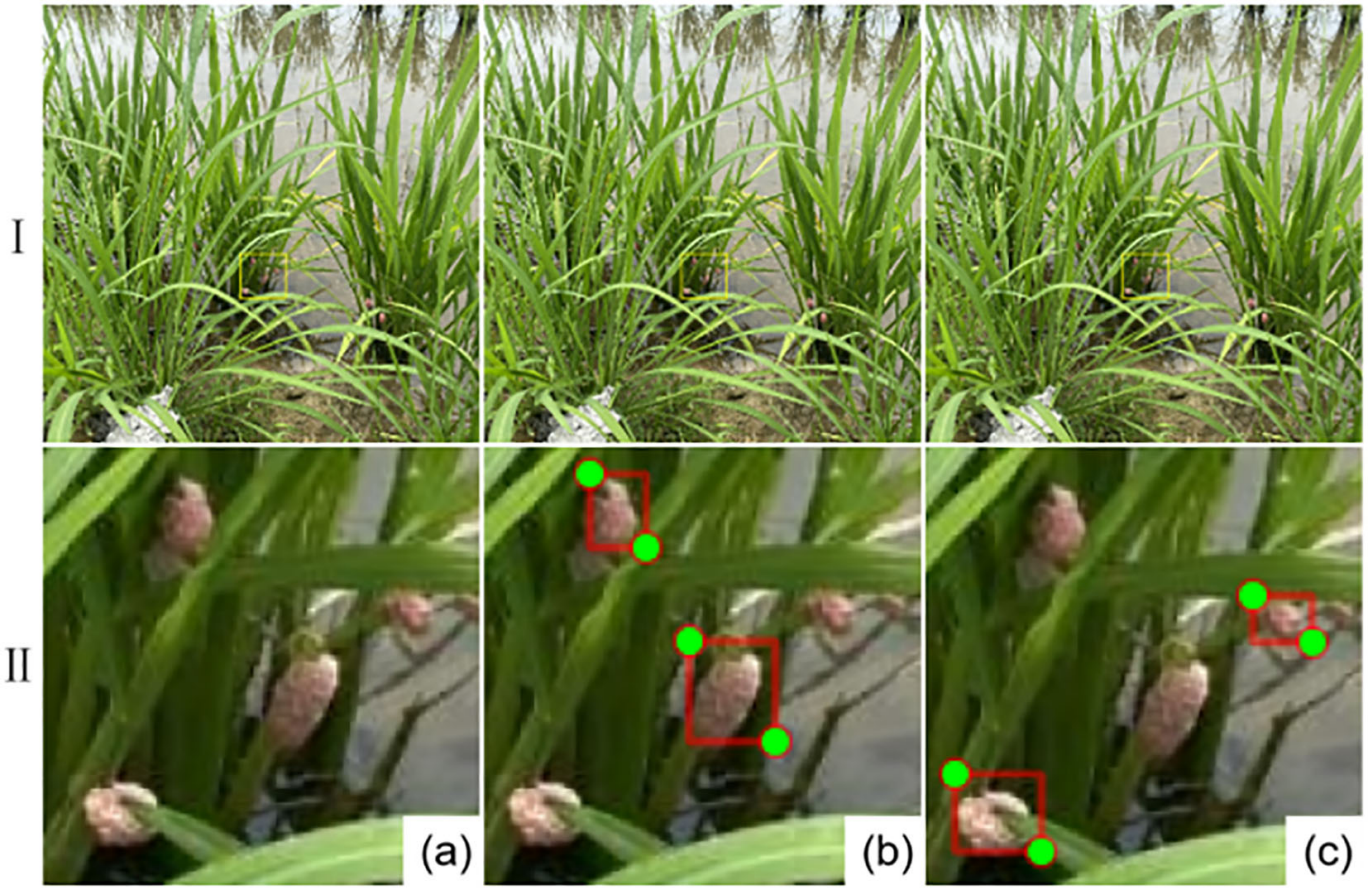
Collected images and label classification results. **(a)** is the original image, **(b)** represents non-occluded eggs, **(c)** represents occluded eggs.

To meet the data requirements of the deep learning network model, all collected images were uniformly resized to 640 × 640 pixels. The data acquired by DJI were cropped into 54 images of 640 × 640 pixels (arranged as 6 rows and 9 columns); the data captured by handheld devices were cropped into 24 images of 640 × 640 pixels (arranged as 4 rows and 6 columns). Meanwhile, data augmentation techniques including rotation, mirroring, Gaussian noise addition, and salt-and-pepper noise injection were applied to the collected images. Operations such as stretching and color transformation were not used, aiming to preserve the shape features of *P. canaliculata* eggs without altering them. This approach not only enriches the dataset but also enhances the generalization and robustness of the model training and the accuracy of target detection. After the augmentation process, a total of 6,783 images were obtained. The Labelme software was used to annotate the features of the images. The annotated *P. canaliculata* eggs were divided into two categories: occluded and non-occluded, to verify the detection performance of the improved model in this paper for occluded *P. canaliculata*. The results of the annotated images and label classification are shown in **Figure 1**, where (A) is the original image, (B) represents non-occluded eggs, and (C) represents occluded eggs. Prior to data augmentation techniques, the dataset was randomly divided into a training set, a validation set, and a test set at a ratio of 8:1:1. This division was performed to ensure that each type of sample was adequately represented in the divided subsets and to prevent augmented variants from the same original region from appearing across different subsets.

### 2.2 Design of the network model

YOLOv8 employs an efficient architecture for feature extraction and object detection, achieving enhanced detection accuracy and accelerated inference speeds. The network comprises four hierarchical components: the input layer for image preprocessing, the backbone layer for feature extraction, the neck layer for multi-scale feature fusion and enhancement, and the head layer for final prediction through classification and bounding box regression (Terven et al., 2023). Specifically, the input layer processes training images, the backbone extracts hierarchical features, the neck integrates contextual information across scales, and the head generates detection outputs by synthesizing semantic and positional features.

**Figure 2:**
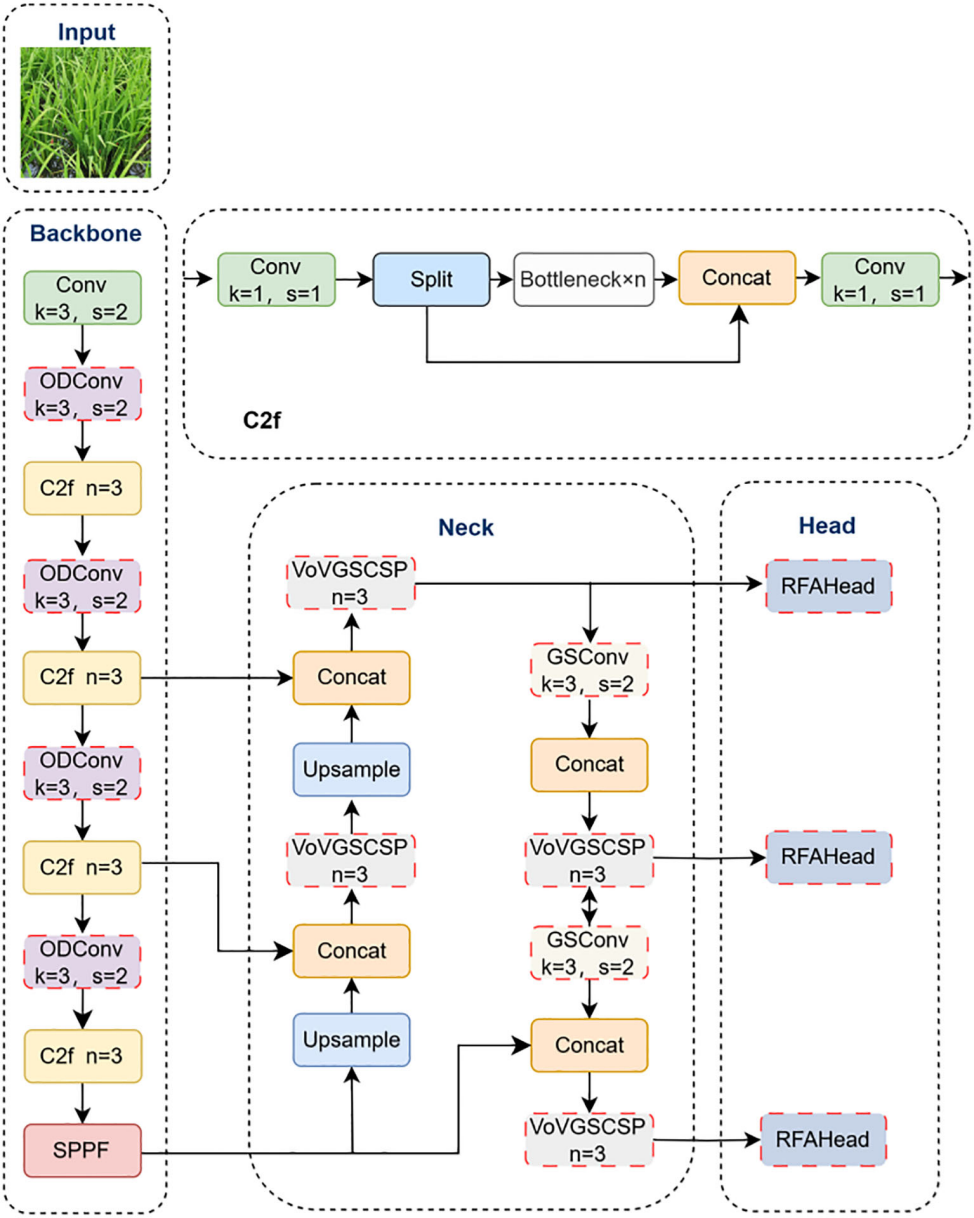
Improved YOLOv8 network structure.

To improve *P. canaliculata* egg detection in complex paddy environments, we propose three targeted modifications to YOLOv8 (**Figure 2**). Firstly, ODConv replaces standard convolutions (except the initial layer) in the backbone, addressing feature representation limitations that cause misdetections while reducing computational complexity. Secondly, the Slim-neck architecture integrates generalized-sparse convolution (GSConv) to optimize feature transmission efficiency and preserve inter-channel dependencies. Complementary VoV-GSCSP modules enhance cross-stage feature fusion, improving detection precision without compromising speed. Finally, the detection head incorporates receptive-field attention convolution (RFAConv), which synergizes spatial attention mechanisms with convolutional operations to prioritize subtle local patterns, particularly for occluded and small-scale targets.

#### 2.2.1 Omni-dimensional dynamic convolution

The mutual occlusion between rice plants and *P. canaliculata* eggs in paddy fields challenges the YOLOv8n model in precisely localizing egg feature regions, resulting in critical identification information loss. To mitigate this limitation, we integrate ODConv into the backbone network, coupled with a multi-dimensional attention mechanism. This hybrid strategy employs parallel learning of complementary attentional weighting across all four convolutional kernel dimensions: spatial size, input and output channel numbers, and kernel quantity (Li et al., 2022b). The synergistic design enhances feature discriminability for occluded targets while reducing computational overhead, thereby improving both detection accuracy and efficiency.

**Figure 3:**
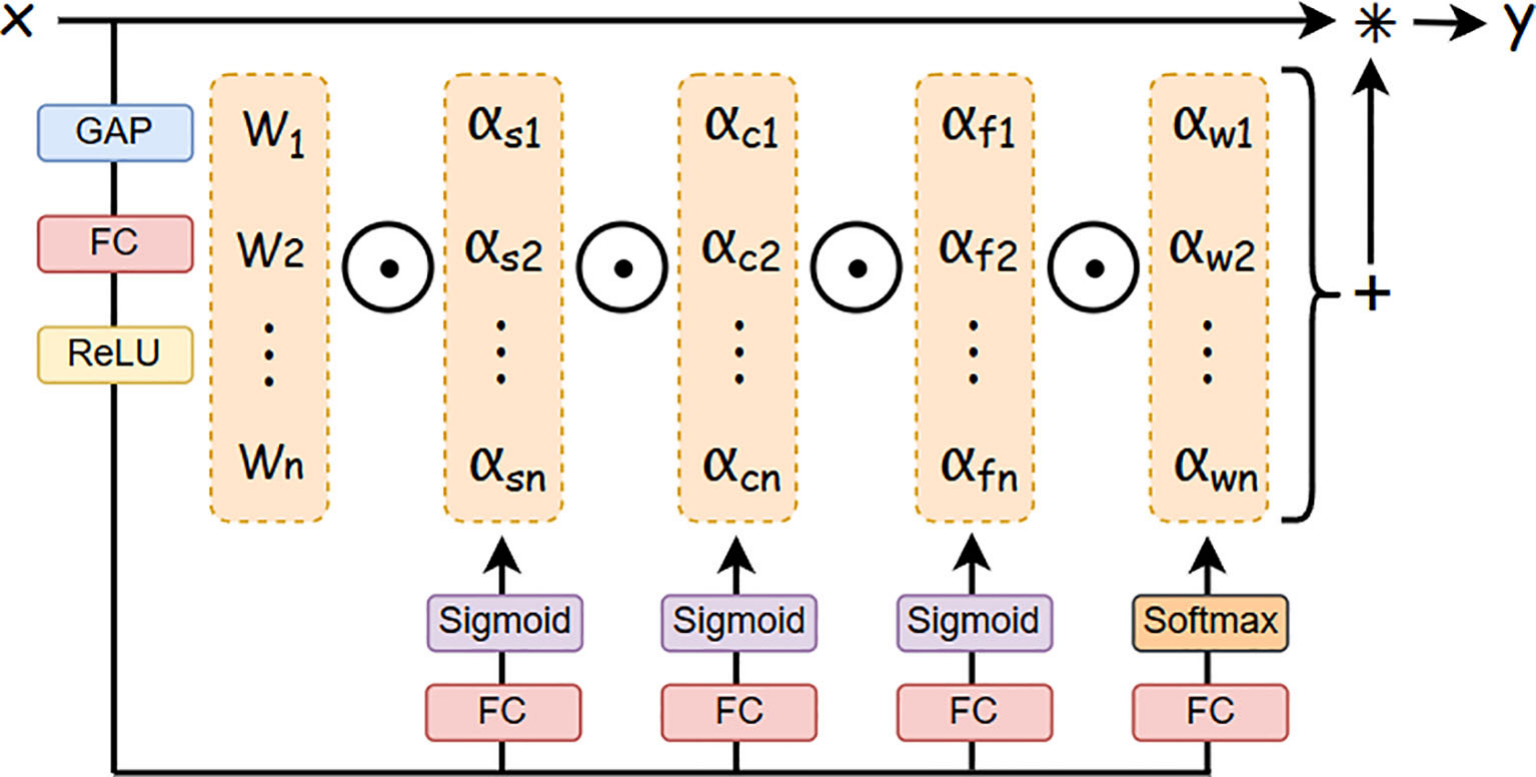
Omni-dimensional dynamic convolution structure.

As illustrated in **Figure 3**, the ODConv architecture operates through three sequential stages. Initially, the input feature map x undergoes dimension reduction via a global average pooling (GAP) layer, producing a $C_{in}$-length feature vector. Subsequently, this vector is processed through fully connected (FC) layers and rectified linear unit (ReLU) activation, generating four parallel attention branches. Each branch incorporates an FC layer followed by softmax/sigmoid normalization, yielding four attention scalars ( $\alpha_{si}$, $\alpha_{ci}$, $\alpha_{fi}$, $\alpha_{wi}$) that dynamically modulate the convolutional kernel $W_{i}$. Finally, the adaptively weighted kernels perform convolution with the input x, synthesizing the output feature map y.

**Figure 4:**
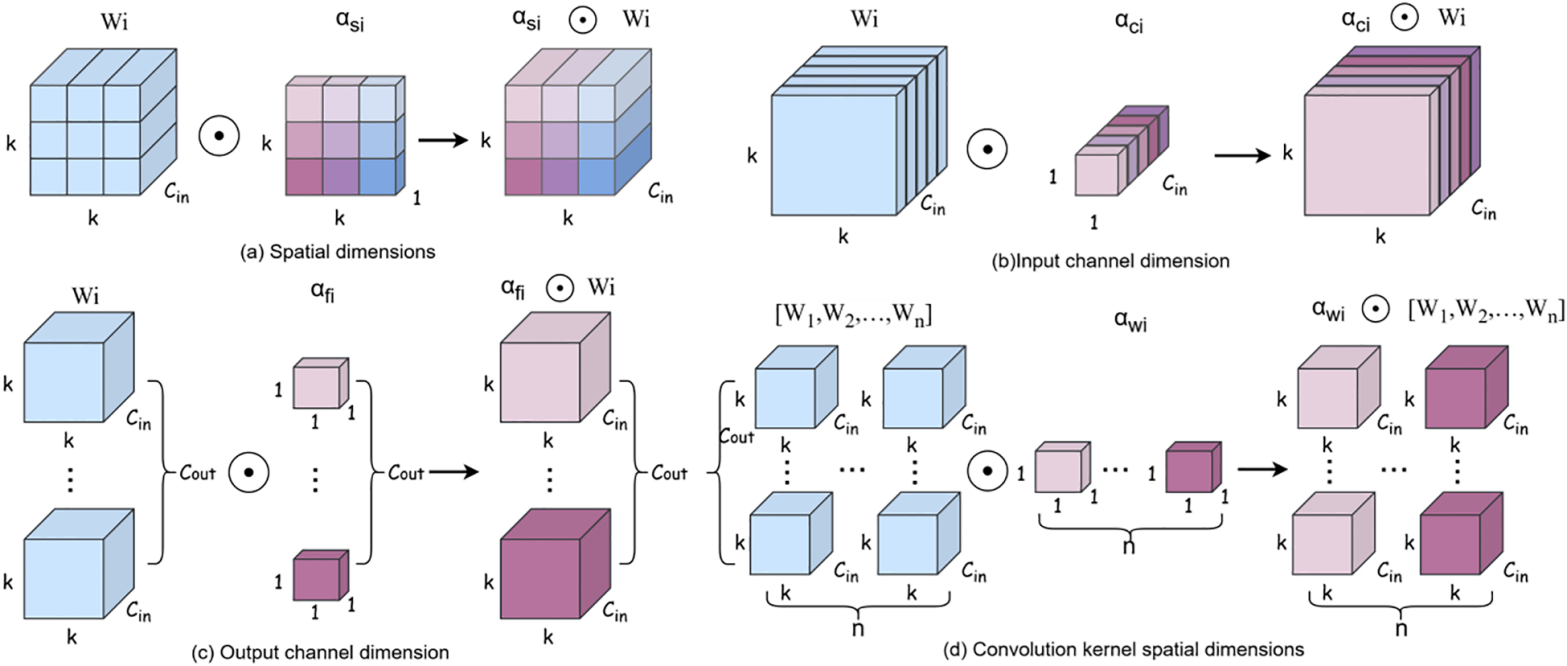
Different attention mechanisms. **(a)** is Spatial dimensions, **(b)** is Input channel dimension, **(c)** is Output channel dimension, **(d)** is Convolution kernel spatial dimensions.

**Figure 4** illustrates the operational mechanisms of four distinct attention branches in ODConv. **Figure 4A** demonstrates spatial-wise element-wise multiplication, where the spatial attention scalar $\alpha_{si}$ computed by ODConv is applied to each spatial position (height and width) of the convolutional kernel. This mechanism enhances the model’s capability to extract spatially discriminative features from *P. canaliculata* egg images, particularly under partial occlusion conditions. **Figure 4B** depicts input channel-wise multiplication, whereby the channel attention scalar $\alpha_{ci}$ dynamically modulates the convolutional kernel across input channels. This adaptive weighting prioritizes occlusion patterns and densely distributed features in egg clusters, thereby improving robustness in complex paddy field environments. **Figure 4C** presents output filter-wise multiplication along the output channel dimension. The filter attention scalar $\alpha_{fi}$ recalibrates the importance of individual output filters, enabling differentiated feature extraction between occluded and non-occluded egg instances. **Figure 4D** implements kernel-wise multiplication, where the kernel attention scalar $\alpha_{wi}$ globally adjusts the entire convolutional kernel. This holistic adaptation allows the model to dynamically optimize kernel parameters for rice field-specific egg characteristics. By replacing standard convolutions (excluding the initial layer) in the backbone with ODConv, our approach synergistically enhances feature extraction across all four dimensions (spatial, input channel, output filter, and kernel space), resulting in quantifiable improvements in detection precision for challenging agricultural scenarios.

#### 2.2.2 Slim-neck structure

**Figure 5:**
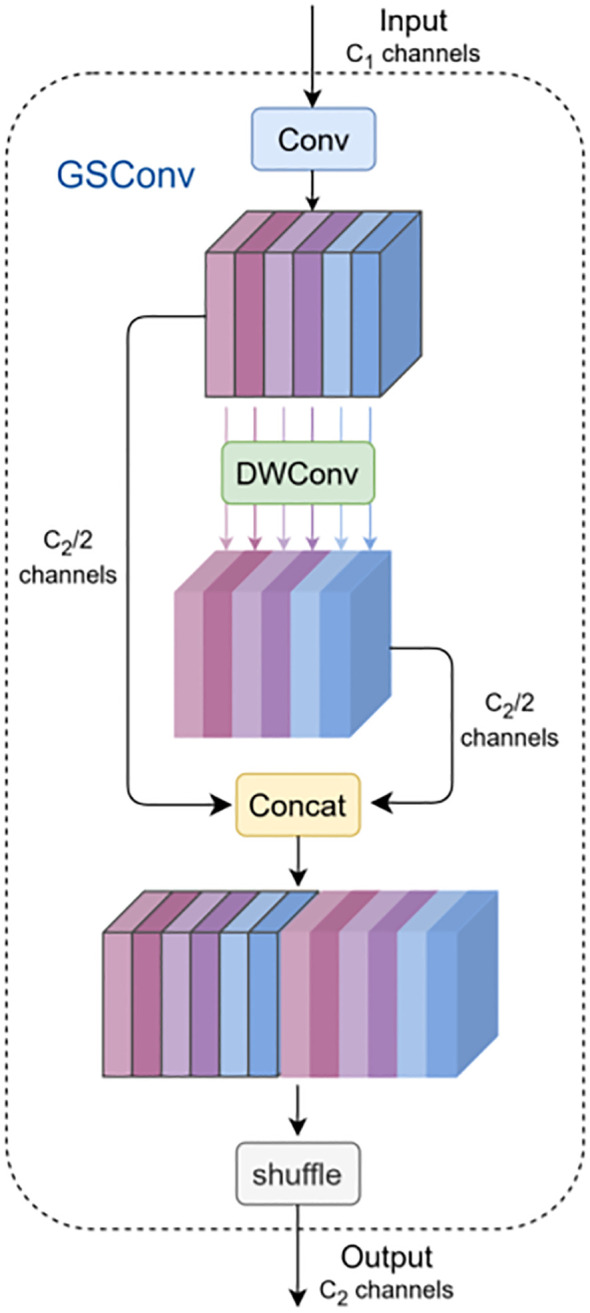
GSConv structure.

The incubation period of *P. canaliculata* eggs ranges from 8 to 16 days (Lv et al., 2024). Deploying detection models on cameras or drones for real-time egg distribution monitoring requires balancing detection accuracy with computational efficiency. To address this, GSConv is introduced to reduce model complexity while improving accuracy, with a Slim-neck structure designed in the neck network (**Figure 5**) (Li H. et al., 2022). The GS bottleneck module enhances feature processing capability, and the VoV-GSCSP module improves feature utilization efficiency, collectively optimizing detection performance for *P. canaliculata* eggs in paddy fields.

MobileNets and ShuffleNets employ depth-wise separable convolutions (DSCs) to accelerate inference but exhibit insufficient accuracy for egg detection (Howard et al., 2017; Zhang et al., 2018). GSConv first applies standard convolution for downsampling. The outputs are then processed by depth-wise convolution (DWConv), concatenated, and shuffled to exchange localized features across channels. This strategy prevents channel information separation during computation, reduces parameters/FLOPs, and preserves interchannel semantic relationships. While spatial compression and channel expansion induce partial semantic loss, dense convolutions retain implicit interchannel connections, whereas sparse convolutions discard them. GSConv partially preserves these connections but increases network depth and inference latency if universally applied. Since the neck stage maximizes channel dimensions and minimizes spatial resolutions, GSConv is selectively implemented only in the neck to minimize semantic information loss.

**Figure 6:**
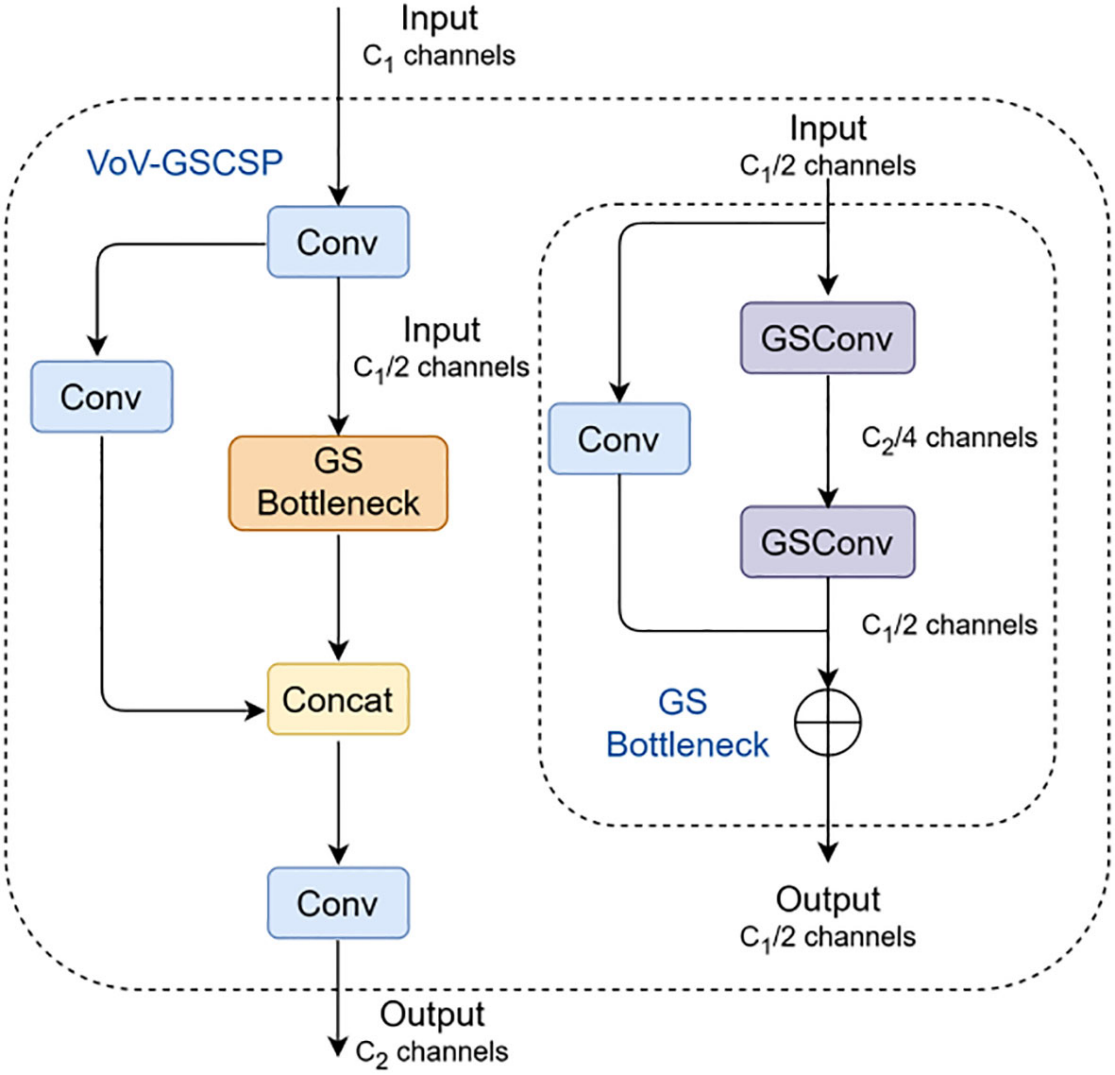
GS bottleneck and VoV-GSCSP structure.

Building upon GSConv, we further introduce GS bottleneck and VoV-GSCSP to reconstruct the neck network of YOLOv8n. Specifically, the original C2f module is replaced with VoV-GSCSP, while standard convolutions are substituted by GSConv, thereby establishing the proposed Slim-neck architecture. The structures of the GS bottleneck and VoV-GSCSP are shown in **Figure 6**, where VoV-GSCSP is designed using a one-shot aggregation strategy. First, a 1 × 1 convolution performs feature extraction on the input, reducing the channel dimension to half of the original input (C_1_ → C_1_/2). Subsequently, the processed features are fed into the GS bottleneck module, where they undergo two GSConv operations and are then combined with features from a parallel 1 × 1 convolution via residual addition, resulting in an output channel dimension of C_1_/2. The original input of the VoV-GSCSP module is separately processed by another 1 × 1 convolution and concatenated with the GS bottleneck output. Finally, a 1 × 1 convolution adjusts the concatenated features to the target channel dimension C_2_.

#### 2.2.3 Receptive-field attention detection head

The standard convolution of the YOLOv8n model applies homogeneous convolutional kernels across all receptive fields, extracting features with identical parameters regardless of spatial location. This design fails to account for position-specific variations in input data, severely limiting the model’s ability to handle multi-scale targets and degrading its feature extraction performance, particularly for small objects such as *P. canaliculata* eggs or partially occluded targets. While attention mechanisms enhance feature discriminability by directing computational resources to salient regions and improving detailed feature capture, conventional modules [e.g., CBAM and coordinate attention (CA)] primarily address spatial feature modeling but inadequately resolve the parameter-sharing limitation of large kernels or emphasize feature importance within receptive fields (Woo et al., 2018; Hou et al., 2021).

**Figure 7:**
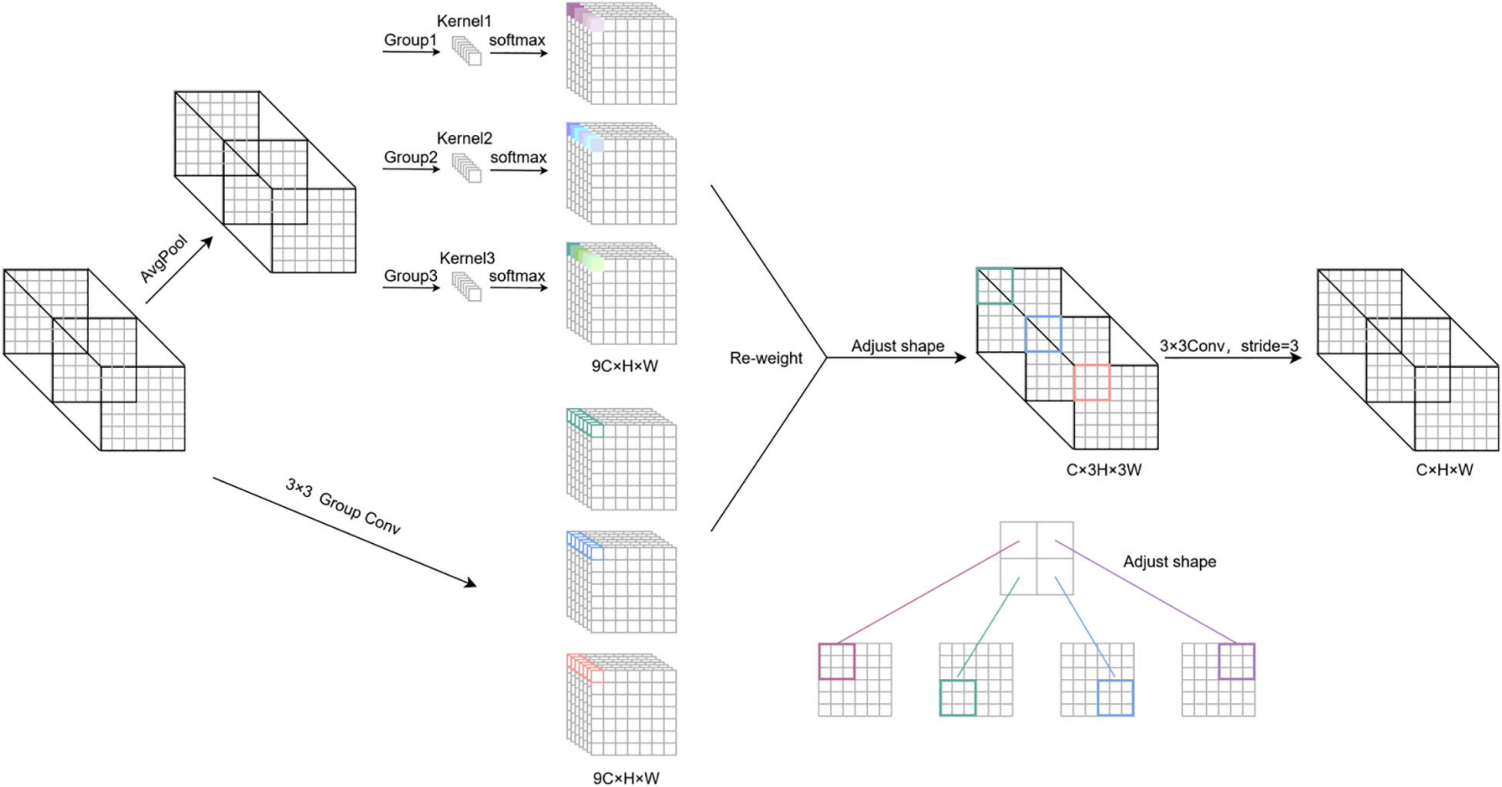
Detailed structure of RFAConv. The input feature map is processed by two branches, and the attention map and the receiving field spatial feature map are obtained, and then, they are reweighted and adjusted to realize the feature extraction of non-shared parameters.

To address the uniform kernel parameterization in the YOLOv8n detection head, this study proposes an RFAHead incorporating RFAConv, as illustrated in **Figure 7**. Let *C*, *H*, and *W* denote the channel number, height, and width of the input feature map, respectively. The RFAConv operates through two parallel branches to obtain weights and spatial features. In the weight branch, global contextual information is aggregated via average pooling (AvgPool), followed by channel interaction using 1 × 1 group convolution. Features are extracted through a receptive field slider to avoid overlap, and a softmax operation dynamically weights feature importance across the receptive field, effectively reducing network parameters without information loss. In the feature branch, the input undergoes 3 × 3 convolution to generate intermediate features with dimensions 9C × h × w. After dimensional transformation, these features are multiplied by the weights to produce the final receptive field feature map (C × 3h × 3w), as detailed in Equation 1.

(1) $$F=Softmax\left(g^{1\times 1}\left(AvgPool\left(X\right)\right)\right)\times ReLU\left(Norm\left(g^{k\times k}\left(X\right)\right)\right)=A_{rf}\times F_{rf}$$

In the formula, $g^{1\times 1}$ represents a grouped convolution with a size of i×i, k represents the size of the convolution kernel, Norm represents normalization, X represents the input feature map, and the output feature F is obtained by multiplying the attention map with the transformed receptive-field spatial features $A_{rf}$ through $F_{rf}$.

**Figure 8:**
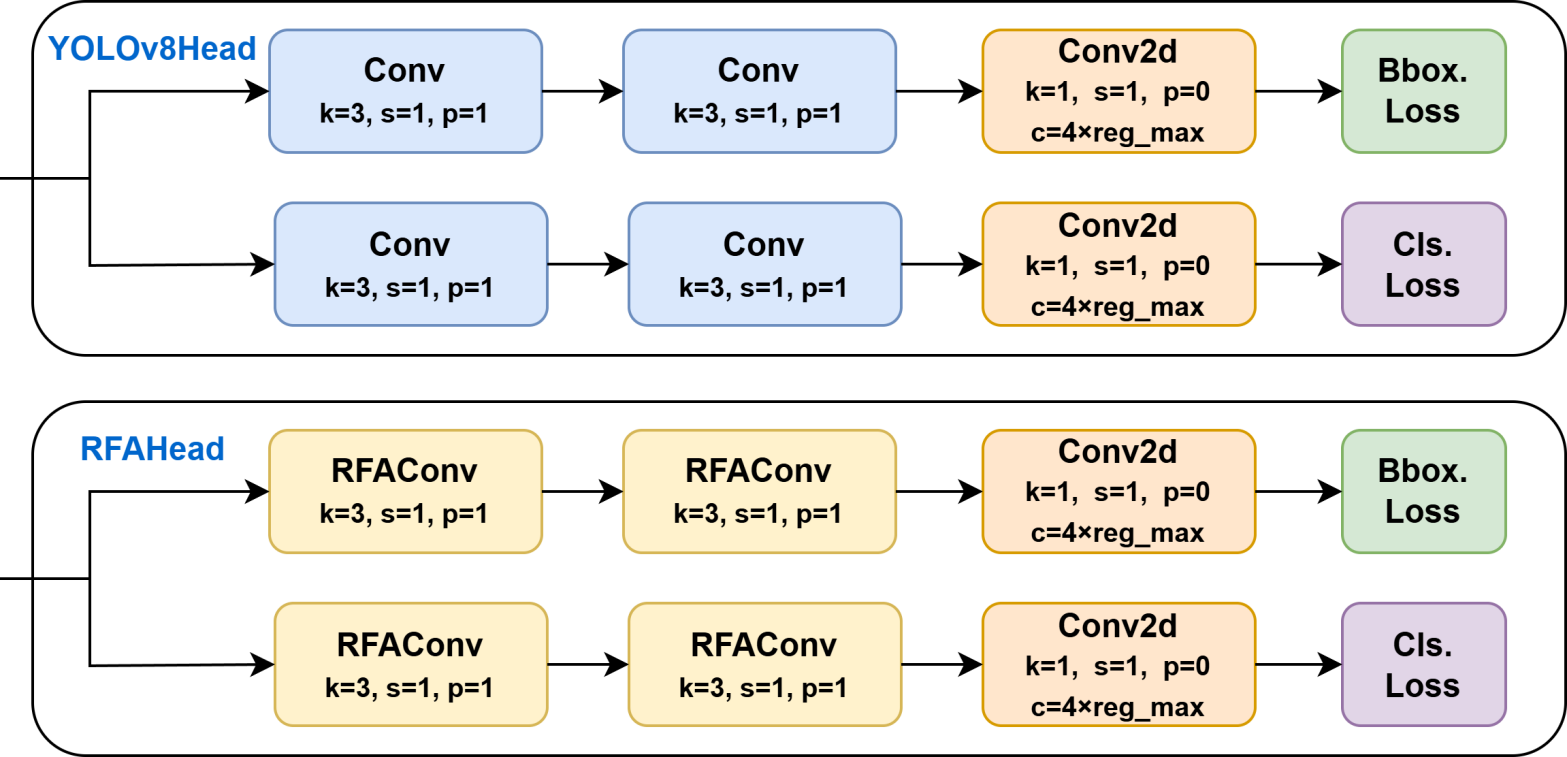
YOLOv8n detection head and receptive field attention detection head structure.

In standard convolution, overlapping receptive field slider features are inevitable, leading to identical attention weights being assigned to shared input features across different receptive fields. Convolutional parameters within each slider should not be fully shared but instead adaptively adjusted based on local feature characteristics and their corresponding attention weights. This adaptation enables the network to process each region with finer granularity, thereby better capturing and responding to input-specific patterns rather than relying on uniform weight application globally. RFAConv dynamically adjusts convolutional kernel weights by adaptively highlighting critical regions in the feature map. Consequently, the network reweights key features, allowing large-sized convolutional kernels to simultaneously capture broad contextual information and focus computational resources on high-informative regions. Such capability enhances processing efficiency and network performance while optimizing feature understanding and representation, ultimately improving learning and predictive accuracy. The structure of the enhanced YOLOv8n detection head (**Figure 8**) resolves the convolutional parameter-sharing limitation and significantly enhances detection precision.

## 3 Experiments and analysis

### 3.1 Experimental environment

The experimental environment was configured as follows: PyTorch 1.13.0, Python 3.9.7, CUDA 11.6.2, and cuDNN 8.6.0. The hardware setup included a Windows 10 system with an NVIDIA GeForce RTX 3050 GPU (8 GB VRAM) and an AMD Ryzen™ 5 6600H processor with Radeon Graphics. To mitigate overfitting and improve generalization, stochastic gradient descent (SGD) was employed as the optimizer. The initial learning rate was set to 0.01 to maintain moderate step sizes for weight updates, with a momentum factor of 0.937 to stabilize gradient descent. Training utilized a batch size of 16 and ran for 200 epochs.

### 3.2 Evaluation indicators

In this study, the recognition accuracy (precision), recall rate, floating-point operations (FLOPs), average detection precision (Average precision), and mAP are used as evaluation indicators to measure the detection performance of the network model for *P. canaliculata* eggs in paddy fields. The calculation formulas for each indicator are as follows:

(2) $$P=\frac{TP}{TP+FP}$$

(3) $$R=\frac{TP}{TP+FN}$$

(4) $$\;AP=\int_{0}^{1}P\left(R\right)dR$$

(5) $$mAP=\frac{1}{N}\sum_{n=1}^{N}AP_{n}$$

In the Equations 2–5, TP denotes the number of correctly detected egg targets; FP represents the number of false positives misclassified as egg targets;  FN indicates the number of undetected egg targets; and AUC (area under the curve) quantifies the area under the precision-recall curve. K  denotes the total number of classes, and APi refers to the average precision for the i-th class. mAP@0.5 represents the mAP across all classes at an IoU threshold of 0.5, while mAP@0.5:0.95 denotes the average mAP computed over IoU thresholds ranging from 0.5 to 0.95 with a step size of 0.05.

### 3.3 Performance comparison

**Figure 9:**
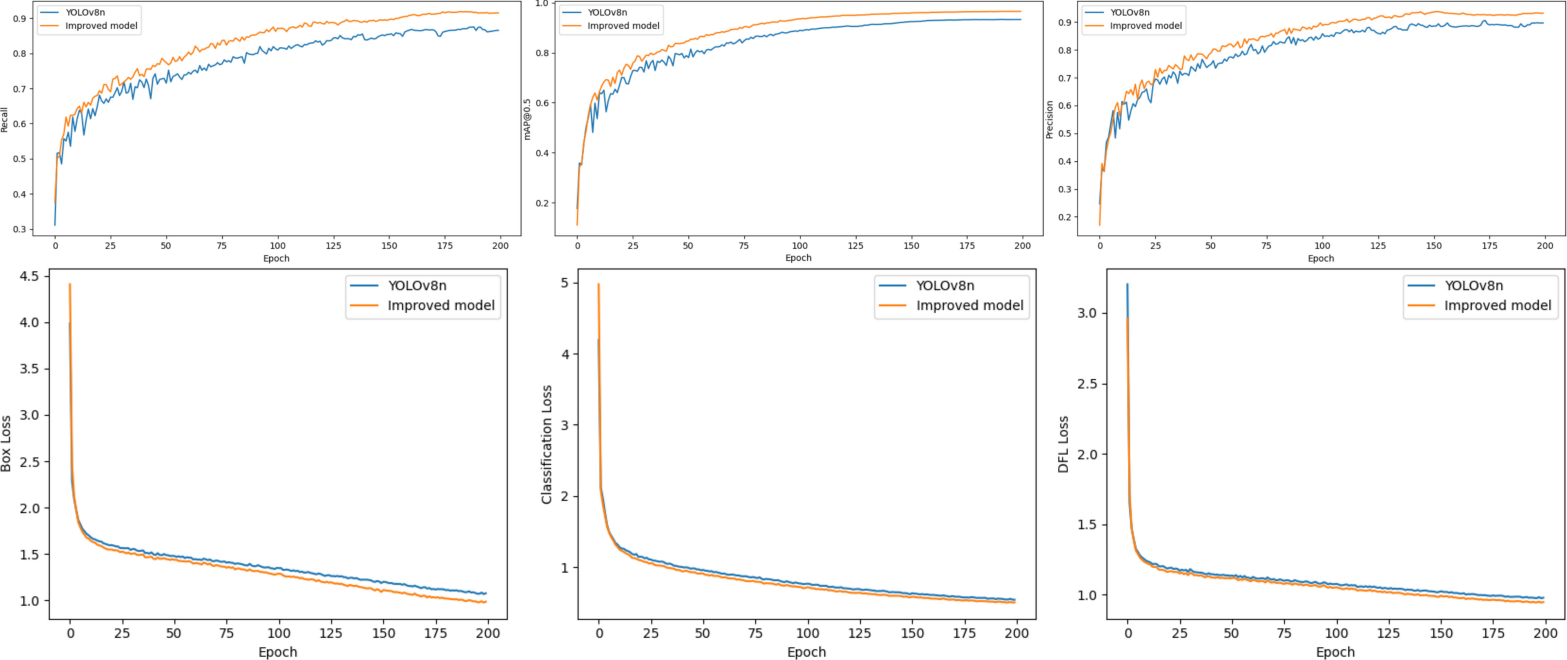
YOLOv8n training curves before and after improvement.

To analyze the detection capabilities of the improved YOLOv8n object detection algorithm for *P. canaliculata* eggs, both the original YOLOv8n and the improved model were trained under identical experimental environments and settings. The training curves of these two models are presented in **Figure 9**.

As shown in **Figure 9**, the blue lines represent the performance metrics of the original YOLOv8n during training, while the orange lines correspond to those of the improved model. Throughout the training process, YOLOv8n exhibited improvements from 24.7% to 89.7% in precision, 31.1% to 87.5% in recall, and 17.8% to 93.2% in mAP@0.5, with reductions from 3.986 to 1.074 in box_loss, 4.189 to 0.546 in cls_loss, and 3.202 to 0.979 in dfl_loss. In contrast, the improved model demonstrated more significant enhancements: precision increased from 17.1% to 93.0%, recall from 37.6% to 91.7%, mAP 0.5from 11.2% to 96.5%, while box_loss decreased from 4.409 to 0.984, cls_loss from 4.972 to 0.508, and dfl_loss from 2.961 to 0.947. These numerical results clearly indicate that the improved model outperforms the original YOLOv8n in both detection accuracy and loss function optimization.

### 3.4 Ablation experiments

To verify the optimization effect of each module on the performance of the *P. canaliculata* egg detection model in paddy fields, an ablation experiment was conducted on each module using YOLOv8n as the baseline network. The results are shown in **Table 1**.

**Table 1:** The results of the YOLOv8n ablation experiments.

Model	P/%%	R/%	mAP@0.5/%	mAP@0.5:0.95/%	*FLOPs*
YOLOv8n (v8n)	89.7	87.5	93.2	63.6	8.1 × 10^9^
v8n+ODConv (ODC)	92.9	90.3	95.4	67.3	7.3 × 10^9^
v8n+SlimNeck (SL)	89.8	89.5	93.8	63.9	7.1 × 10^9^
v8n+RFAHead (RF)	90.6	88.9	94.5	65.5	8.4 × 10^9^
v8n+ODC+Sl	92.7	91.5	95.3	67.2	6.4 × 10^9^
v8n+ODC+RF	92.5	90.8	95.5	67.5	7.6 × 10^9^
v8n+Sl+RF	91.8	88.0	94.4	64.9	7.5 × 10^9^
v8n+ODC+Sl+RF	93.0	91.7	96.5	68.4	6.7 × 10^9^

While maintaining the original architecture of YOLOv8n, the integration of ODConv significantly improved both precision and recall rates compared to the baseline model. Specifically, the mAP 0.5and mAP@0.5:0.95 metrics increased by 2.2% and 3.7%, respectively. This enhancement demonstrates that ODConv strengthens the model’s ability to extract features of *P. canaliculata* eggs by learning complementary attention across four dimensions, thereby improving detection accuracy. Incorporating the Slim-neck structure into the neck network reduced computational complexity by 1.0 GFLOPs while marginally improving precision, recall, mAP@0.5, and mAP@0.5:0.95. The VoV-GSCSP module and GSConv not only enhanced feature processing capabilities but also optimized computational efficiency, which is critical for real-time detection of *P. canaliculata* eggs in field environments. Replacing the original detection head with the proposed RFAHead increased mAP@0.5 and mAP@0.5:0.95 by 1.3% and 1.9%, respectively, with precision and recall rising by 0.9% and 1.4%. However, this modification incurred a computational cost increase of 0.3 GFLOPs, attributable to RFAConv’s adaptive kernel weight adjustment during feature extraction, which prioritizes informative regions. As shown in **Table 1**, the three enhancement strategies—ODConv, Slim-neck, and RFAHead—exhibit synergistic effects without mutual interference. The combined model (v8n + ODC + Sl + RF) achieved improvements of 3.3% in precision, 4.2% in recall, 3.3% in mAP@0.5, and 4.8% in mAP@0.5:0.95 over the baseline, alongside a computational reduction of 1.4 GFLOPs. These results validate that the proposed modular improvements collectively enhance both accuracy and reliability for detecting *P. canaliculata* eggs in paddy field environments.

### 3.5 Comparison experiments of different models

Since the targets of *P. canaliculata* eggs are relatively small and there are often occlusion situations in the real field environment (Huang et al., 2023), aiming at the problems of dense distribution of eggs and poor recognition effects when there is occlusion, this paper has improved the structural network of YOLOv8n. In order to further verify the performance and effectiveness of the detection model among mainstream networks, the network model proposed in this paper is compared experimentally with Faster R-CNN (Ren et al., 2017), YOLOv3-tiny (Adarsh et al., 2020), YOLOv5n, YOLOv6n (Li et al., 2022a), YOLOv7-tiny (Cheng et al., 2023), YOLOv8n, YOLOv9-t (Wang C et al., 2024), YOLOv10n (Wang A et al., 2024), and YOLOv11n (Khanam and Hussain, 2024). The experimental results are shown in **Table 2**. All training processes were configured using the parameters of the improved model to avoid the influence of parameters on training results. Specifically, SGD was adopted as the optimization strategy during the model optimization process, with an initial learning rate set to 0.01 and a momentum factor of 0.937 to stabilize the gradient descent process. The training was conducted with a batch size of 16 and ran for 200 epochs, where the key hyperparameters were set as follows: lr0 = 0.01, lrf = 0.01, mosaic = 1.0, box = 7.5, fliplr = 0.5, and flipud = 0.1.

**Table 2:** Comparison test of different models’ results.

Model	*P/%*	*R/%*	*mAP@0.5/%*	*mAP@0.5:0.95/%*	*FLOPs*	*FPS*
Faster R-CNN	81.4	75.7	78.3	47.8	1.1 × 10^11^	36.8
YOLOv3-tiny	83.7	75.5	84.1	50.9	1.9 × 10^10^	121.1
YOLOv5n	90.1	86.4	91.3	60.3	7.1 × 10^9^	71.9
YOLOv6n	84.5	78.2	85.7	52.8	1.2 × 10^10^	102.2
YOLOv7-tiny	85.6	80.3	84.9	52.3	1.3 × 10^10^	116.4
YOLOv8n	89.7	87.5	93.2	63.6	8.1 × 10^9^	99.7
YOLOv9-t	87.7	86.5	91.5	61.4	6.5 × 10^9^	90.5
YOLOv10n	86.2	84.6	92.7	60.7	6.3 × 10^9^	112.9
YOLOv11n	90.3	86.4	93.1	61.9	6.3 × 10^9^	105.3
Ours	93.0	91.7	96.5	68.4	6.7 × 10^9^	113.7

The improved model in this study has the highest values in mAP@0.5 and mAP@0.5:0.95, reaching 96.5% and 68.4% respectively. Compared with Faster R-CNN, YOLOv3-tiny, YOLOv5n, YOLOv6n, YOLOv7-tiny, YOLOv8n, YOLOv9-t, YOLOv10n, and YOLOv11n, the mAP@0.5 of the improved model is 18.2, 12.4, 5.2, 10.8, 11.6, 3.3, 5.0, 3.8, and 3.4 percentage points higher, respectively, and the mAP@0.5:0.95 is 20.6, 17.5, 8.1, 15.6, 16.1, 4.8, 7.0, 7.7, and 6.5 percentage points higher, respectively. The precision and recall rates also reach 93.2% and 91.7%, which are higher than those of other mainstream networks. Its computational amount reaches 6.7G, which is only higher than that of YOLOv10n and YOLOv11n (6.3G each). The detection frame rate reaches 113.7 frames per second, which is higher than that of YOLOv5n, YOLOv7n, YOLOv8n, YOLOv9-t, YOLOv10n, and YOLOv11n, but slightly lower than that of YOLOv3-tiny and YOLOv7-tiny, meeting the requirements for deploying the device on mobile terminals for real-time detection. Since Faster R-CNN has a two-stage network structure and the feature maps extracted by the convolutional extraction network are all single-layer maps with relatively small resolutions, its accuracy and FPS are lower than those of the YOLO series networks, while the computational amount is large. The comparison experiments with different models further prove the effectiveness of the improvement strategy and the superiority of the improved model in the task of detecting *P. canaliculata* eggs in paddy fields.

### 3.6 Embedded deployment and optimization

Although personal computers and servers possess powerful training and inference capabilities, their large size, high power consumption, and limited portability limit their applicability to object detection tasks that require lightweight platforms and real-time processing (Dong et al., 2025a). As an embedded deployment platform, NVIDIA Jetson Nano features a small size, low power consumption, and high computational efficiency, making it highly suitable for edge vision analysis in resource-constrained environments (Dong et al., 2025b).

In this study, the improved YOLOv8n model was deployed on the Jetson Nano B01 (4 GB) embedded device, and comparative verification was conducted against the original YOLOv8n model to evaluate the improvement in frames per second (FPS) of the improved model. During the deployment process, the TensorRT inference engine was used for fusing and simplifying the network structure, achieving a trade-off between accuracy and speed. The FPS of the original YOLOv8n model on Jetson Nano reached 4.98, which fails to meet the requirements of real-time detection tasks. After optimization with TensorRT, the FPS of the improved YOLOv8n model reached 48.60, which verifies the feasibility of deploying the improved model on mobile terminals. The comparative results before and after optimization are presented in **Table 3**.

**Table 3:** Jetson Nano inference comparison.

Model	Parameter (M)	FPS (Jetson Nano)	GFLOPs
Yolov8n	4.1	4.98	8.1
Ours	3.0	48.60	6.7

### 3.7 Visualization analysis

**Figure 10:**
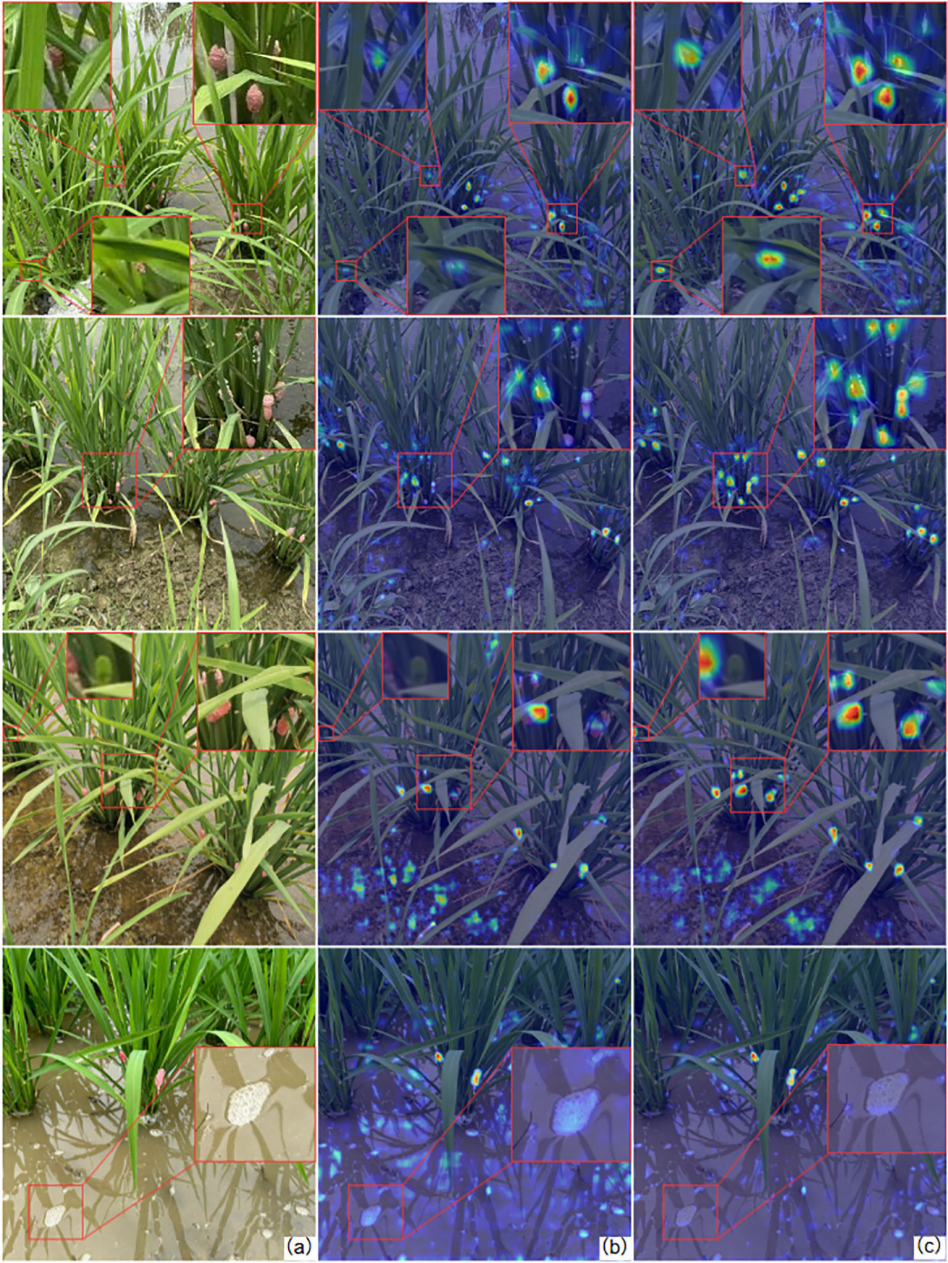
Comparison of the heatmap between the YOLOv8 model and the improved model. **(A)** Original image; **(B)** the YOLOv8n model; **(C)** the improved model.

In this study, gradient-weighted class activation mapping (Grad-CAM) (Selvaraju et al., 2017) was employed to visualize the feature activation patterns of egg detection. The visualization results for layer 22 (detection head layer) of the model are presented in **Figure 10**. Grad-CAM is a deep learning-based interpretability method that highlights image regions critical for model predictions. In the first case, the baseline YOLOv8n model shows negligible attention to heavily occluded *P. canaliculata* eggs. After optimization, the enhanced model demonstrates improved extraction of detailed textures from occluded eggs, enabling accurate recognition. In the second case, the original model fails to activate features of small-sized and densely distributed *P. canaliculata* eggs, leading to missed detections. The modified network significantly strengthens attention to these challenging targets. In the third case, the baseline model exhibits insufficient semantic understanding of image boundaries, ignoring edge-located *P. canaliculata* eggs while over activating background artifacts (e.g., water surface impurities). The improved architecture integrates global context to enhance spatial feature aggregation, thereby boosting edge-target detection accuracy and suppressing interference. In the fourth case, the baseline model exhibited false detections on water surface scum and air bubbles. The improved detection head, integrated with the receptive field attention mechanism, enables more refined detection of local regions, thereby resolving the false detection issue of water surface air bubbles.

**Figure 11:**
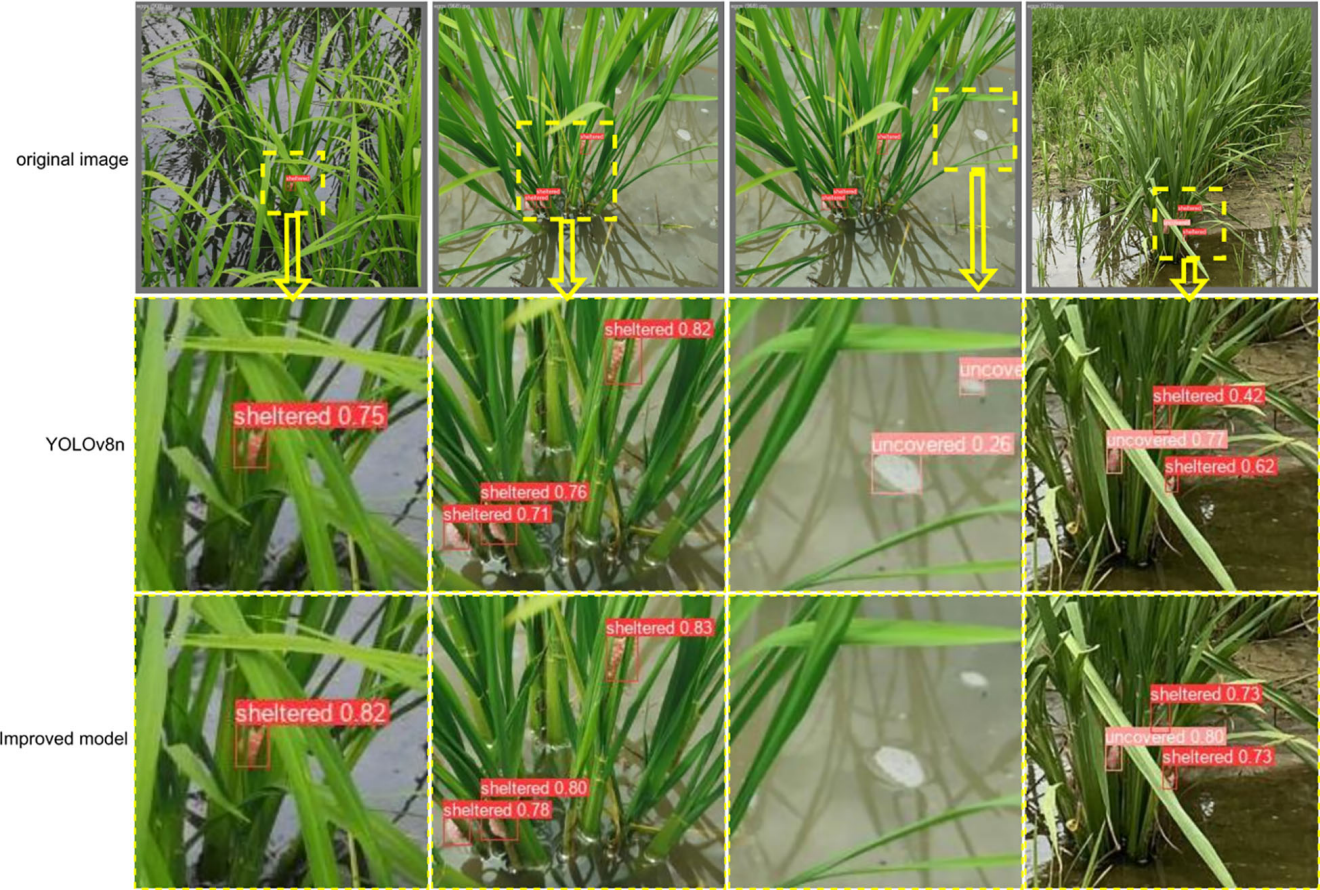
Comparison of detection effect before and after model improvement.

**Figure 11** compares the detection performance of the baseline and improved YOLOv8n models. The enhanced model exhibits significantly higher confidence in detecting small, occluded *P. canaliculata* egg targets within complex paddy field environments. In columns 1–2, the improved model demonstrates increased confidence for partially occluded eggs compared to the baseline. Column 3 reveals that the original YOLOv8n generates low-confidence false positives on water surface foam, whereas the optimized model eliminates such errors. Column 4 highlights a substantial confidence improvement for both heavily occluded and non-occluded eggs in the enhanced model.

These results, combined with the heatmap analysis, confirm that the improved model strengthens attention to small and occluded targets while mitigating false and missed detections. The visualization validates the efficacy of the proposed dataset construction and augmentation strategies in boosting model performance.

### 3.8 Generalization experiment

To further verify the generalization performance of the improved YOLOv8n model, the publicly available dataset released by Professor Ningzhong Liu from Nanjing University of Aeronautics and Astronautics was selected for testing in this section. The dataset was collected via multi-angle low-altitude photography using a DJI drone (FC2220) at Taihu Lake (119°11′–121°53′E, 30°08′–32°08′N), and its access link is https://drive.google.com/file/d/1lOUIUelA6mdmBrr2WswQrHjDf6edt9Xu/view?usp=sharing. Each image in the dataset has a size of 1,000 × 800 pixels. The images cover various common scenarios to ensure higher robustness of the model. A total of 5,000 images were selected for testing, and the test results are presented in **Table 4**.

**Table 4:** Improved YOLOv8n model generalization results.

Model	*P/%*	*R/%*	*mAP@0.5/%*	*mAP@0.5:0.95/%*
YOLOv5n	81.4	74.9	81.3	46.5
YOLOv8n	81.3	75.5	83.2	47.3
YOLOv10n	79.8	73.4	80.7	44.7
YOLOv11n	82.0	75.8	82.1	46.5
Improved YOLOv8n	85.3	75.7	84.5	50.9

The experimental results demonstrate that the improved YOLOv8n model outperforms other benchmark models in key detection metrics for *P. canaliculata* eggs. Compared with YOLOv8n, its mAP@0.5 and mAP@0.5:0.95 are increased by 1.3 and 3.6 percentage points, respectively. These results not only confirm the improved robustness of the model but also highlight the enhanced generalization performance through the more stringent mAP@0.5:0.95 metric.

## 4 Conclusions

To achieve rapid and precise identification of *P. canaliculata* egg clusters in complex paddy ecosystems, this study proposes an optimized YOLOv8n model incorporating three key innovations: ODConv, Slim-neck architecture, and RFAHead. These modifications collectively address critical challenges including false positives, missed detections, and occlusion issues, significantly enhancing detection accuracy and model robustness. Key findings are summarized as follows:

1. First, we replace standard convolutions with ODConv in the backbone network, leveraging multi-dimensional attention mechanisms to amplify feature extraction capabilities for occluded and small-sized eggs. Second, the neck network is redesigned by substituting C2f modules with VoV-GSCSP and standard convolutions with GSConv, forming a Slim-neck structure that improves feature utilization efficiency. Finally, we engineer RFAHead with optimized convolutional kernels, integrating receptive field attention mechanisms. These synergistic modifications reduce computational complexity by 18% while boosting detection precision, achieving real-time performance suitable for edge devices.
2. A dedicated dataset comprising 6,783 annotated *P. canaliculata* egg images was constructed through systematic field collection and data augmentation techniques. Ablation studies confirm the efficacy of each modification, with the enhanced YOLOv8n achieving state-of-the-art performance: 96.5% mAP@0.5 and 68.4% mAP@0.5:0.95, at 113.7 FPS with 6.7 GFLOPs computational load. Comparative trials demonstrate 12.3% higher accuracy than baseline models under occlusion scenarios.
3. Visualization of detection results demonstrates that the improved model effectively identifies *P. canaliculata* eggs in complex paddy field environments, showing high-precision detection capabilities in tests using naturally acquired images. In future studies, we will expand experimental samples and integrate transfer learning to enhance the model’s generalization ability, further optimizing detection accuracy and real-time performance. The model will be tested on mobile devices to enable timely egg detection and implement corresponding control measures, providing data and methodological references for rice pest management.

The improved YOLOv8n model proposed in this study has achieved relatively satisfactory detection performance on the self-built *P. canaliculata* dataset, but there are still some limitations. Restricted by the current data collection conditions, the dataset is insufficient in terms of sample size and environmental complexity (e.g., water quality, meteorological conditions, substrate composition, etc.). In subsequent studies, we will continuously expand the data scale and enhance data diversity to improve the model’s adaptability in different practical scenarios. To enhance the deployment efficiency and portability of the model in practical applications, we will also introduce lightweight optimization strategies such as model pruning and knowledge distillation. On the basis of ensuring detection accuracy, we will further compress the model size and reduce computational resource consumption and hardware dependence. Future work will focus on the deployment tests of the model on edge devices or mobile terminals and evaluate its real-time performance and stability in field environments.

## Data Availability

The raw data supporting the conclusions of this article will be made available by the authors, without undue reservation.
